# ‘Bringing services to communities: Identifying service users at risk of developing cardiovascular disease through mobile opportunistic screening in deprived or underserved communities’

**DOI:** 10.1016/j.puhip.2025.100690

**Published:** 2025-12-13

**Authors:** Jill West, Matthew Humphreys, Marianne Durand, Emma Green, Kevin D. Hochard, Alex Stewart

**Affiliations:** a Cheshire and Wirral Partnership NHS Foundation Trust, Chester Health Park, Chester, CH2 1BQ, UK; b Division of Psychology, University of Chester, CH1 4BJ, UK; c UK Health Security Agency, Liverpool, UK

**Keywords:** Healthcare inequalities, Health inequity, Service access, Lifestyle risk factors, CVD, Operational research

## Abstract

**Objective:**

Individuals in areas of high deprivation face significant health inequalities with a higher prevalence of risk factors for cardiovascular disease (CVD). Whilst some factors are non-modifiable, early identification of modifiable factors and appropriate intervention can improve health outcomes. We assessed modifiable risk factors, particularly in persons with no medical history.

**Study design:**

A retrospective cross-sectional study using data of persons attending a mobile opportunistic screening clinic.

**Method:**

Data was extracted from the records of 2973 attendees (≥18 years) in Cheshire & Merseyside, January–June 2023 using a standard approach. We classed CVD risk factors as either modifiable or non-modifiable. We grouped the data into those with no risk factors, one or two, and three or more, in relation to their self-reported medical history or clinical findings.

**Results:**

Within the total study population of 2973, 79 % had at least one modifiable risk factor for CVD. Our logistic regression model highlighted that non-modifiable factors age and sex were significant predictors of CVD, while males had higher odds than females to report CVD. Of our modifiable factors, only hypertension and mental health diagnosis were significant predictors. BMI was excluded from the multivariable analysis due to missing data.

**Conclusion:**

Mobile screening provides improved equitable access to services and engages with underserved communities to deliver targeted health care. It identifies CVD risk in an asymptomatic population, and patients with poorly controlled conditions. This model is highly acceptable to service users and is flexible and targeted in its activities and placement.

## 1 What this study adds

As a community-based study, it provides real world data which can be shared with other agencies to develop/tailor services to address locality needs and demonstrates that providing outreach mobile health screening to targeted communities offers the opportunity for more equitable access.

## 2 Implications for policy and practice

This study highlights the opportunities mobile outreach services offer to those who have difficulty accessing mainstream services. However, relationships with local health care providers/resources need to be established/developed to ensure robust follow up strategies are in place for patients identified as requiring additional support/intervention.

Targeted service offerings in underserved communities require multiagency collaboration and input if they are to support the social, physical and emotional needs of the communities they serve.

## 3 Introduction

Cardiovascular disease (CVD) is an umbrella term covering ischaemic heart disease and stroke as well as several other less common vascular diseases. In the United Kingdom, CVD currently affects around seven million people and accounts for one in four premature deaths [1,2]. Two major causes of death from CVD are ischaemic heart disease and stroke causing 460 deaths every day from ischaemic heart disease, with approximately 130 of these being in people under 75 years of age. Stroke (cerebrovascular disease) causes about 93 deaths every day, with 274 new strokes daily and is the biggest cause of severe disability in the country [3].

Numerous risk factors for CVD are well documented. Risk factors can be categorised in different ways: as modifiable and non-modifiable; related to co-morbidities; and environmental, social and biological. Risk factors which can be modified include smoking, weight, and hypertension whilst risk factors such as age or sex are non-modifiable. Co-morbidities are commonly found in service users with CVD and include high cholesterol, diabetes, chronic hepatic, renal, respiratory disease, dementia, and mental health conditions. The National Institute for Health & Care Excellence (NICE) advocate risk assessment for those aged 40–74 years of age, who do not have a diagnosis of CVD, diabetes mellitus, or chronic kidney disease, to identify those most at risk of developing CVD [4].

Individuals in the most deprived areas of England face significant healthcare inequalities affecting access to healthcare, healthcare experience and disease patterns [5,6]. Other factors such as economic stability, social support networks, where people are born, access to education and other broader social issues in their locality also impact health outcomes. Lower socio-economic groups tend to have a higher prevalence of higher risk health behaviours or risk factors, such as poor diet, smoking, and reduced access to health care, and have less opportunity to lead healthier lives than higher socio-economic groups [7]. As a result, there is often a higher incidence of obesity, cardiovascular disease, cancer, and mental health issues [1]. One in five adults with low-income report having difficulty accessing health care services and delay accessing services e.g. Emergency Departments [8]. Results from a poll reported that people avoid vital health services due to fear over costs [9].

The Vaccination and Living Well Service is a mobile outreach health screening service in Cheshire and Merseyside (North West England) where many of these risk factors are highly prevalent [3,10,11]. The Vaccination and Living Well Service adopts a life-course approach; the vision is to optimise every social and clinical contact, making every contact count, with the aim of improving health and social well-being with age specific (≥18 years), targeted interventions to members of deprived communities who struggled to access services.

The Vaccination and Living Well Service provides targeted health care assessment and non-pharmaceutical intervention(s) in areas of high deprivation, low healthcare engagement or where inequity of access is a known barrier. Opportunistic screening is offered to anyone 18 years and older, irrespective of their medical history. Data is collected for modifiable and non-modifiable risk factors. Clinical, social and psychological interventions are actioned as appropriate.

The Living Well Service is funded through a combination of NHS and local authority funds, which are allocated to Health Service Commissioners and social care budgets to support community-based services. Staffed by trained health care professionals, its three mobile units deliver targeted health care services throughout Cheshire and Merseyside.

Although outside of the scope of this study the service also offers COVID-19 vaccination, during seasonal campaigns, routine immunisations to the general population from eight weeks of age and single out-patient COVID treatment. The Living Well service developed out of a realisation that the vaccination service was reaching underserved communities.

Little is known about whether such mobile screening services are effective in identifying people at risk of CVD. The study aimed to understand the clinical and demographic characteristics of service users who presented opportunistically for health screening and associated risk of developing CVD. Identification of relevant characteristics will enable the service to work with identified agencies to offer appropriately targeted interventions to improve CVD outcomes, particularly in underserved areas.

## 4 Method

### 4.1 Setting

Cheshire and Wirral Partnership NHS Foundation Trust (CWP) provides health and care services for local people, including mental health, learning disability, community physical health and all-age disability care, together with the provision of community services including General Practices.

### 4.2 Study design

This was a retrospective cross-sectional study using data collected from service users who attended for opportunistic health screening January–June 2023. Data was extracted anonymously from paper records.

The Vaccination and Living Well Service developed clinical pathways, with the Local Medical Council (LMC) in Cheshire, adapted from NICE Guidelines, to define markers for CVD risk factors. [12,13]^,^ These included hypertension, cholesterol, BMI, pulse check, and capillary blood glucose measurement. Whilst random capillary blood glucose is not currently part of the national screening programme, it is used as part of opportunistic health screening programmes in under-represented communities with increased diabetes risk to detect pre-diabetic states [14]. Risk markers for blood pressure, cholesterol, BMI, glucose, pulse and mental health pathways were graded, red, amber and green, with those with red markers (excluding BMI as a single marker) requiring urgent referral or review (Table 1).

**Table 1:** Classification of risk according to clinical parameters

Classification of risk according to clinical parameters							
(Adapted from NICE Guidelines, to define markers for CVD risk factors [12,13])							
						**Mental Health Pathways**	
						
						
**Classification**	**Blood Pressure**	**Total cholesterol**	**Capillary blood glucose**	**Body Mass Index**	**Alcohol units per week**	**PHQ-9**	**GAD-7**
**Red**	180/120 mmHg	>7.5 mmol/l	>11.1 mmol/l	>40	≥20	≥20	≥15
**Amber**	179/119–141/91 mmHg	5.0–7.5 mmol/l	7.8–11.0 mmol/l	25-39 or <18.5	15–19	10–19	8–14
**Green**	<140/90 mmHg	<5.0 mmol/l	<7.7 mmol/l	18.5–24.9	≤14	≤9	≤7

Deprivation data was derived from patient postcodes [15]. The white ethnicity rate in Cheshire and Merseyside was calculated form ONS data from the 2021 census [16,17].

### 4.3 Study population

The study population included everyone aged 18 years and over, resident in any of the nine local authorities in Cheshire and Merseyside, who accessed the outreach service for opportunistic health screening, irrespective of any known clinical conditions, from January to June 2023.

### 4.4 Data collection and validation

Data was extracted from 2936 paper clinical records. The data was cleansed, removing records which had incomplete data for ≥5 of the 20 data points, service users not registered with a GP, or who refused all tests. As the intention is to undertake further work to examine the outcomes for patients who are referred to primary care, those patients not registered with a GP who had a clinical review had their findings excluded from the data as we were unable to locate for follow-up.

The final dataset consisted of 2793 service user records. Thereafter, data validation was undertaken by an independent reviewer on a 2.5 % sample, resulting in an overall correction rate of 2.3 %.

### 4.5 Analysis

We identified demographic and clinical characteristics, modifiable and non-modifiable risk factors. We used numbers, percentages and Chi square to identify relevant patterns. The data was coded and analysed using open-source statistical software (Jamovi version 2.7). To assess the relative influence of factors on CVD risk, a hierarchical logistic regression was performed to predict CVD (yes/no) including non-modifiable factors; sex, age, ethnicity and deprivation index decile in the first block. The second block entered into the regression contained modifiable factors; alcohol intake, smoking status, diabetes status (yes/no), hypertension status (yes/no), and mental health diagnosis (yes/no). Pseudo-R^2^ (Cox and Snell, McFadden, Nagelkerke), odds ratios and 95 % confidence intervals were obtained.

## 5 Results

Examination of the demographic and clinical characteristics of the service users in the study population highlighted several important modifiable and non-modifiable risk factors for CVD (Table 2).

**Table 2:** Demographic and clinical characteristics of service users attending for mobile opportunistic health screening (n = 2793 unless otherwise noted).

Characteristic	Detail	n	%
**Non-modifiable risk factors**			
Sex	Male	1241	44.4
	Female	1542	55.2
Age (years)	18–39	523	18.7
	40–69	1645	58.9
	≥70	625	22.4
Ethnicity (n = 2576)	White	2186	84.9
	Non-white	390	15.1
**Modifiable risk factors**			
Smoking (n = 2754)	Vaper	87	3.2
	Current smoker	373	13.5
	Ex-smoker	521	18.9
	Never smoked	1773	64.4
Alcohol (units/week) (n = 2761)	0	1113	40.3
	1–5	1152	41.7
	6–10	179	6.5
	11–15	151	5.5
	>15	166	6.0
Substance use (n = 2189)	Never	2125	97.1
	Cannabis	30	1.4
	Heroin	3	0.1
	Cocaine	12	0.6
	Other	19	0.9
Deprivation quintile	1 (most)	1293	46.3
	2	403	14.4
	3	366	13.1
	4	432	15.5
	5 (least)	299	10.7

The age range of the study population was 18–97 years; 60 % of attendees (1645) were between 40 and 69 years of age. Ethnicity was predominantly white (85 %) but more non-white attendees were seen (15 %) than predicted from the local population non-white (6 %).

The 2022 GP Patient Survey for England highlighted that men make fewer general practice appointments than women [18] and this was mirrored in our findings by the higher number of women (55.2 % v 44.4 %) attending for opportunistic screening than men, with most attendees coming from the areas with the highest level of deprivation (almost 60 %). There were low levels of smoking (82 % were ex- or non-smokers), alcohol (81 % reported drinking 5 units or less per week) and substance use (76 % reporting no history of substance use, although 22 % of the data for this group was either missing or declined) reported by the attendees.

For those with no previous history (self-reported) the analysis identified attendees who were asymptomatic and yet had raised markers e.g., high cholesterol, for developing CVD. For diabetes 11.3 % of those with no history had raised markers for diabetes, for hypertension 25 %, cardiac 38 % and for BMI it was 65 %; it should be noted that 20 % of attendees declined to have BMI measured or data was missing and this resulted in BMI being excluded from the multivariable analysis. For mental health conditions, of the 77 % who reported no history almost 6 % had raised scores with 2.5 % scoring for depression and over 3 % reporting symptoms of anxiety when using validated assessment tools, PHQ-9 and GAD-7 respectively [19,20] (Table 3).

**Table 3:** Past medical history and modifiable risk factors identified.

	Diabetes		Hypertension		Cardiac		BMI		All mental health		Mental Health PHQ-9		Mental Health GAD-7	
	N	%	n	%	n	%	n	%	n	%	n	%	n	%
Cohort	2793	100.0	2793	100.0	2793	100.0	2232	79.9	2793	100.0	2162	100.0	2162	100.0
History of	238	8.5	504	18.0	229	8.2	N/A		542	19.4				
No previous history	2491	89.2	2215	79.3	2477	88.7	N/A		2162	77.4				
Missing/declined	64	2.3	74	2.6	87	3.1	561	20.1	89	3.2	1105	51.0	1101	50.0
aAmber/red MRF	281	11.3	546	24.7	930	37.5	1437	65.0	99	4.6	44	2.0	55	2.5
aRed MRF	39	1.6	34	1.5	23	0.9	88	6.1	27	1.3	10	0.5	17	0.8
Not clinically indicated	**-**		–		–		–		–		861	39.8	858	39.7

a No previous history recorded yet had 1 or more amber/red MRF recorded at assessment. Amber and red risk markers are used to grades of severity and are Adapted from NICE guidelines. N/A - not applicable.; MRF - modifiable risk factor. PHQ-9 is a Depression scoring tool. GAD-7 is an Anxiety scoring tool.

Our hierarchical logistic regression to predict CVD categorisation is reported in Table 4. Block 1 which included non-modifiable factor (age, sex, ethnicity, IMD decile) accounted for 2.5 %–7.4 % of variance in CVD categorisation (*R*^*2*^_*CS*_ = 0.06, *R*^*2*^_*McF*_ = 0.03, *R*^*2*^_*Nag*_ = 0.07, *Χ*^*2*^[4] = 28.98, *p* < .001). The inclusion of modifiable factors (alcohol intake, smoking status, diabetes status, hypertension status, and mental health diagnosis) in block 2 increased total variance explained estimates to between 4.8 % and 14.1 % (*R*^*2*^_*CS*_ = 0.05, *R*^*2*^_*McF*_ = 0.12, *R*^*2*^_*Nag*_ = 0.14, *Χ*^*2*^[14] = 56.08, *p* < .001).

**Table 4:** Hierarchical logistic regression predicting CVD (yes/no) from non-modifiable and modifiable factors.

						95 % Confidence Interval	
Predictor	Estimate	SE	Z	p	Odds ratio	Lower	Upper
Intercept	−7.49	1.31	−5.74	<0.001	0.00	0.00	0.01
Age	0.07	0.02	3.67	<0.001	1.08	1.04	1.12
Gender:							
Male – Female	0.63	0.28	2.22	0.026	1.88	1.08	3.28
Ethnicity:							
Non-White – White	−0.80	0.52	−1.53	0.125	0.45	0.16	1.25
Deprivation index	−0.03	0.05	−0.61	0.540	0.97	0.88	1.07
Alcohol:							
1–5 units – Non-Drinker	−0.54	0.32	−1.71	0.087	0.58	0.31	1.08
6–10 units – Non-Drinker	−0.25	0.53	−0.48	0.630	0.78	0.28	2.18
11–15 units – Non-Drinker	−0.98	0.77	−1.27	0.203	0.37	0.08	1.70
>15 units – Non-Drinker	−0.89	0.58	−1.54	0.123	0.41	0.13	1.27
Smoking:							
Smoker – Non-Smoker	0.11	0.41	0.27	0.790	1.11	0.50	2.47
Ex-Smoker – Non-Smoker	−0.04	0.36	−0.11	0.910	0.96	0.47	1.94
Vaper – Non-Smoker	0.64	0.69	0.93	0.350	1.90	0.50	7.27
Diabetes:							
Diabetes – No Diabetes	0.67	0.37	1.84	0.066	1.96	0.96	4.02
Hypertension:							
Hypertension – No Hypertension	0.80	0.29	2.71	0.007	2.22	1.25	3.95
Mental Health Diagnosis:							
Yes – No	0.63	0.30	2.08	0.037	1.88	1.04	3.41

Note. Estimates represent the log odds of "Heart disease = CVD" vs. "Heart disease = No CVD".

The data highlighted several notable differences: One third of the non-white population had no modifiable risk factors as opposed one fifth in the white community. Greater than two thirds of adults aged 40 years or over, had one or more modifiable risk factors whilst around half of younger people (18–39) have one or two modifiable risk factors (54 %). Females had lower odds of having 0–2 risk factors whilst males had higher odds of 3 or more modifiable risk factors (Table 5).

**Table 5:** Number of modifiable risk factors by age, gender, deprivation and ethnicity (n = 2793).

			Number of modifiable risk factors						Chi square	*P*
	Service users		0		1–2		≥3			
	N	%	n	%	n	%	n	%		
**Age in years**									103.8	<0.001
18-39	523	18.7	192	36.7	284	54.3	47	9.0		
40-69	1645	58.9	283	17.2	1088	66.1	274	16.7		
70 plus	625	22.4	107	17.1	424	67.8	94	15.0		
**Gender**									9.3	0.001
Male	1241	44.4	249	20.1	780	62.9	212	17.1		
Female	1542	55.2	331	21.5	1011	65.6	200	13.0		
Missing	10	0.4								
**Ethnicity**									32.8	<0.001
White	2186	78.3	412	18.9	1435	65.7	339	15.5		
Non-white	390	14.0	120	30.8	233	59.7	37	9.5		
Missing/declined	217	7.8								
**Deprivation Quintiles**									18.2	0.020
1 (most)	1293	46.3	252	19.5	827	64.0	214	16.6		
2	403	14.4	96	23.8	261	64.8	46	11.4		
3	366	13.1	86	23.5	236	64.5	44	12.0		
4	432	15.5	91	21.1	265	61.3	76	17.6		
5 (least)	299	10.7	57	19.1	207	69.2	35	11.7		

## 6 Discussion

Within the asymptomatic population presenting for opportunistic screening across Cheshire and Merseyside, the study identified a high level of modifiable risk factors that could be altered, or in fact negated, thereby reducing the risk of the service user developing CVD. It also identified non-modifiable risk factors that impact the risk of CVD e.g., sex. Service users with non-modifiable risk factors for CVD such as age, ethnicity or sex also had modifiable risk factors that can be altered to reduce the overall risk of CVD. This has implications for service delivery and multi-agency working as not all interventions may be pharmacologically led.

Invitations for the national CVD health check programme start at forty years of age, with the NHS Health Check offering 15 million persons, aged 40–74 years a health check every five years [21], yet the study suggests that modifiable risk factors exist in people younger than forty, with over 60 % in the 18–39 group having one or more modifiable risks. It would seem likely that early intervention, or in the case of the national programme earlier intervention, could with pharmacological or non-pharmacological support and/or behavioural change, alter the trajectory of CVD disease progression. The mobile screening service offers an opportunity to identify CVD risk in younger people not eligible for the national screening programme who are clearly interested in their health status. As expected, there was a higher number of women attending for opportunistic screening than men.

Determining whether more people use mainstream health services or mobile screening services in localities is complex, as it varies significantly by location, population, services offered locally, accessibility and cultural barriers such as language and health beliefs. We believe the very specific, localised targeting of our services to areas of known deprivation or poorly resourced/underserved communities is reflected in our findings. For Cheshire and Merseyside, regional data for white ethnicity is 94 % [16]. Data from the study demonstrates whilst most service users were white, there was a higher percentage of non-white service users (15 %) than expected from the regional figures (6 %), demonstrating that the service reaches ethnic minorities, improving equitable access for those underserved communities. There was a high level of engagement with service users in areas of high deprivation, over 50 % in IMD deciles 1–4, which reinforces the success of the targeted placement of the mobile buses in these areas and the acceptance of the service in these communities.

In the 18–39 age group 38.5 % reported a mental health condition (21 % and 8.8 % respectively for the other two age groups). Whilst not totally unexpected, it may reflect the unprecedented demands upon young people today living in areas of high deprivation and has significant implications for mental health resource and services if this issue is to be tackled effectively.

The most significant outcome from the study was the number of asymptomatic service users the study identified with one or more modifiable risk factors for CVD in the amber and red zones. A raised BMI was recorded in over 65 % of the service users, this was also the highest data set for missing or declined data. In a report by NHS England lifestyles team nationally around two thirds of adults were overweight or obese, with more men than women being recorded as obese or overweight (68 % v 60 %), with adults living in the most deprived areas most likely to be obese [21]. This is a modifiable risk factor; however, to alter this requires behavioural change, with or without pharmacological or non-pharmacological support and an understanding of dietary choices by the service user. Other more complex interactions such as genetics, the environment we live in which influences diet and activity levels, socioeconomics including our ability to purchase healthy food options, the emotional drivers for that individual that cause overeating, the timing and engagement of planned interventions are some of the other important factors that need to be considered to effect change. Whilst this result was not unexpected, it may present an opportunity for more targeted service offerings in the future. In asymptomatic service users, risk markers for hypertension were recorded in around 25 % and for cholesterol about 37 %; again, both are modifiable risk factors for CVD, with early detection enabling early intervention.

Exploring the relative importance of CVD risk factors, our logic regression model highlighted that non-modifiable factors age and sex were significant predictors of CVD. Namely, that for each additional year, risk of CVD categorisation increased by 8 % (OR 1.08, 95 % C.I. 1.04–1.12), while males had higher odds (OR 1.88, 95 % C.I. 1.08–3.28) than females to report CVD. Of our modifiable factors, only hypertension and mental health diagnosis were significant predictors. Participants reporting having Hypertension were 122 % more likely to have CVD [OR 2.22, 95 % C.I. 1.25–3.95]) than non-hypertensive participants. Similarly, have a mental health diagnosis conferred increased risk (88 % [OR 1.88, 95 % C.I. 1.04–3.41]) compared to non-diagnosed participants. This is in keeping with known factors and indicates that the mobile opportunistic screening clinic identifies a population at risk yet open to reducing risk factors.

### 6.1 Limitations of the study

Some of the data were self-reported and, therefore, may have been under or over-reported e.g. alcohol, or substance abuse, for fear of being judged, or concerns regarding the data being shared with primary care (service users are made aware of this at the start of the consultation). The exclusion of individuals not registered with a GP, who could be argued are the most vulnerable, may introduce an element of selection bias however, the intention was to conduct further research to determine the outcomes of any follow-up received which would not have been possible for these individuals and their data was therefore not included. The point of care testing tools for cholesterol and blood glucose levels only gave basic results (total cholesterol and capillary blood glucose) which are appropriate for opportunistic screening but may have led to over reporting of risk markers. More sophisticated point of care testing, measuring for example HDL/LDL ratio for cholesterol and screening for HbA1c to detect diabetes, would have provided more robust markers. Data collection, variations in clinical interpretation and recording on paper-based assessment sheets, at point of care, may have resulted in inconsistencies in data recording and missing data.

‘Missing’ or ‘declined to provide’ data was significantly higher for some key variables such as BMI (20 %), mental health (51 %) and substance use (22 %). Most of ‘missing’ data was ‘declined to provide’ rather than ‘not recorded’ and this in itself opens up the opportunity for further research into the reasons behind the individual's reluctance to answer. The authors acknowledge the unknown effects these missing data, particularly BMI, might have on the overall results.

There was no data available to determine the outcome of any referral to primary care, other agencies or any intervention made by the health care team at the point of care, and this is recognised as a limitation of the study. It does however, present an opportunity for further research to be undertaken.

### 6.2 For consideration

The use of an electronic database to provide a QRISK score (QRISK3 is a validated algorithm which calculates risk of myocardial infarction or stroke over the next ten years) is currently in development [22].

This will enable more robust, consistent risk calculation and will, using health diagnostic algorithms, tailor assessment and diagnostic tests based on history thus reducing cost and improving service efficiency and effectiveness. This will ensure the service user only has the tests that are clinically indicated and will provide more robust evidence for referral to other agencies. In this study a significant number of attendees declined to provide their alcohol consumption and/or BMI, this will impact any tool used (QRISK, AUDIT-C or other) and may lead to information bias and inaccurate findings.

### 6.3 Conclusion

With primary care services often difficult to access, an ageing population, diverse underserved communities, and other socioeconomic and political considerations we must look at innovative and cost-effective ways to deliver healthcare services and health promotion, to reduce the burden on health, and maximise existing resources. The study demonstrates that the mobile outreach screening service model works. It improves equitable access to services, engages with underserved communities to deliver targeted health care offerings. It identifies CVD risk in an asymptomatic population. It identifies patients with known medical conditions that are not well controlled, it is an acceptable model of health care to the service users who engage and can be flexible and targeted in its activities and placement of services. It is easily replicated, flexible and adaptable and working collaboratively with relevant agencies/services could be used to deliver a number of innovative health screening services across the region in the future.

## Authors’ contributions statement

All authors contributed equally to: (1) the conception and design of the study, or acquisition of data, or analysis and interpretation of data, (2) drafting the article or revising it critically for important intellectual content, (3) final approval of the version to be submitted.

## Ethical statement

This project was approved by Clinical Directors at Cheshire and Wirral Partnership NHS Foundation Trust, as with all studies being conducted at the Trust. This study analyses retrospective data which was collected routinely as part of standard clinical care. Staff have access to this data as part of their clinical duties. All data was anonymised and was not used for any different purpose other than that for which it was collected. There are therefore no ethical or confidentiality issues requiring NHS research approvals. This study is not defined as research, according to the Health Research Authority algorithm (see http://www.hra-decisiontools.org.uk/research/), and was reviewed and approved in the same way as evaluations.

## Funding

This project was conducted through the Structured Operational Research and Training Initiative (SORT IT), a global partnership led by the 10.13039/100016285Special Programme for Research and Training in Tropical Diseases at the 10.13039/100004423World Health Organization (WHO/TDR). The training is based on a course developed jointly by the International Union Against Tuberculosis and Lung Disease (The Union) and Medécins sans Frontières (MSF). This specific SORT IT program was run by Cheshire and Wirral Partnership (CWP) NHS Foundation Trust as part of routine work. The SORT IT course that enabled this project was funded by CWP 10.13039/100030827NHS Foundation Trust. The project itself did not receive any specific grant from funding agencies in the public, commercial, or not-for-profit sectors.

## Declaration of competing interest

The authors declare that they have no known competing financial interests or personal relationships that could have appeared to influence the work reported in this paper.

## Data Availability

Data cannot be shared for ethical/privacy reasons.
